# Zebrafish *ambra1a* and *ambra1b* Knockdown Impairs Skeletal Muscle Development

**DOI:** 10.1371/journal.pone.0099210

**Published:** 2014-06-12

**Authors:** Tatjana Skobo, Francesca Benato, Paolo Grumati, Giacomo Meneghetti, Valentina Cianfanelli, Silvia Castagnaro, Martina Chrisam, Sabrina Di Bartolomeo, Paolo Bonaldo, Francesco Cecconi, Luisa Dalla Valle

**Affiliations:** 1 Department of Biology, University of Padova, Padova, Italy; 2 Department of Molecular Medicine, University of Padova, Padova, Italy; 3 Department of Biology, University of Tor Vergata, Rome, Italy; 4 Department of Neuroscience, Istituto di Ricovero e Cura a Carattere Scientifico “Santa Lucia Foundation”, Rome, Italy; 5 Unit of Cell Stress and Survival, Danish Cancer Society Research Center, Copenhagen, Denmark; National University of Singapore, Singapore

## Abstract

The essential role of autophagy in muscle homeostasis has been clearly demonstrated by phenotype analysis of mice with muscle-specific inactivation of genes encoding autophagy-related proteins. Ambra1 is a key component of the Beclin 1 complex and, in zebrafish, it is encoded by two paralogous genes, *ambra1a* and *ambra1b*, both required for normal embryogenesis and larval development. In this study we focused on the function of Ambra1, a positive regulator of the autophagic process, during skeletal muscle development by means of morpholino (MO)-mediated knockdown and compared the phenotype of zebrafish Ambra1-depleted embryos with that of *Ambra1* ^gt/gt^ mouse embryos. Morphological analysis of zebrafish morphant embryos revealed that silencing of ambra1 impairs locomotor activity and muscle development, as well as *myoD1* expression. Skeletal muscles in ATG-morphant embryos displayed severe histopathological changes and contained only small areas of organized myofibrils that were widely dispersed throughout the cell. Double knockdown of *ambra1a* and *ambra1b* resulted in a more severe phenotype whereas defects were much less evident in splice-morphants. The morphants phenotypes were effectively rescued by co-injection with human *AMBRA1* mRNA. Together, these results indicate that *ambra1a* and *ambra1b* are required for the correct development and morphogenesis of skeletal muscle.

## Introduction

Autophagy is an evolutionarily conserved catabolic process in which cells, through the lysosomal machinery, degrade and recycle long-lived proteins and dismantle organelles in order to maintain a homeostatic intracellular environment. This process is tightly regulated and plays several important roles in normal physiology, differentiation, embryo development, and cell survival during starvation [1]. Defects of this degradative system play a role in various diseases, such as neurodegenerative and lysosomal storage disorders and in oncogenesis and cancer progression [2]. However, little is known about autophagy in muscular pathology.

In skeletal muscle, the role of autophagy was initially demonstrated in *Atg5* and *Atg7* muscle-specific knockout mice [3], [4]. In both models, the muscle showed abnormal mitochondria and disorganized sarcomeres, confirming a homeostatic role of autophagy in this tissue. A direct connection between autophagy deregulation and muscular dystrophy was initially found in collagen VI null mice, where accumulation of abnormal organelles and spontaneous apoptosis was shown to strictly depend on defective autophagy regulation [5]. In agreement with this, reactivation of autophagy restored myofiber survival and ameliorated the dystrophic phenotype of collagen VI null mice. More recently, deregulation of the autophagic process was also demonstrated in other dystrophic mouse models [6]–[9] as well as in the Vici syndrome, a human genetic disease caused by recessive mutations of the *EPG5* gene, which codes for a key autophagy regulator involved in the formation of autolysosomes [10].

Ambra1, originally identified in a gene trap screening in mice, is a positive regulator of the Beclin 1 dependent programme of autophagy [11]. Ambra1 is an intrinsically disordered protein, whose capability of binding a number of other regulatory partners involved in many cell processes highlights its crucial role as a “relay” molecule for autophagy [12]. In mammalian cells, Ambra1 is normally docked at a specific cytoskeletal site, corresponding to the dynein light chain, where it is unleashed upon autophagy induction to translocate at the autophagosome origin sites on the endoplasmic reticulum [13]. Ablation of Ambra1, as demonstrated by loss-of-function in mice, leads to embryonic lethality and causes neural defects, suggesting a role for autophagy in nervous system development [11]. Results obtained with the zebrafish model confirmed the involvement of this protein in embryonic development and demonstrated that the duplicated fish *ambra1* paralogous genes are required for normal embryogenesis and larval development. Indeed, MO-induced ablation of the corresponding proteins was found to be associated with several developmental abnormalities and decreased viability [14].

The rapid development and transparency of zebrafish embryos, together with the high fecundity and amenability to genetic manipulation of this vertebrate model, as well as with the feature that skeletal muscles represent a large portion of the body and are easily accessible for analysis, have made this organism attractive for investigating muscle development and fiber-type specification (reviewed by [15]) as well as myopathies and muscular dystrophies (reviewed by [16]). In this study, we investigated the role of Ambra1 in skeletal muscle development by means of knockdown of *ambra1* paralogous genes in zebrafish. Depletion of zebrafish Ambra1 proteins results in abnormal locomotor activity and a severe myopathy characterized by irregular myofiber orientation and highly disorganized sarcomeres, suggesting a role for Ambra1 in muscle development. In agreement with this, histological analysis of mouse *Ambra1* gene trap mutant (*Ambra1* ^gt/gt^) embryos showed a disorganized three-dimensional structure of developing muscles and an increased proliferation of muscle cells.

## Materials and Methods

### Animal maintenance and handling

Zebrafish (AB strain) were raised, staged and maintained according to standard protocols [17], [18]. Embryos were obtained by natural spawning and cultured in zebrafish fish water solution (50x: 25 g Instant Ocean, 39.25 g CaSO_4_, 5 g NaHCO_3_ for 1 l) at 28.5°C with a photoperiod of 14 h light/10 h dark. For *in vivo* imaging, embryos were anesthetized with 0.04% tricaine [18]. The touch-evoked motor behaviour was stimulated by touching the embryo with a thin tip. *Ambra1* ^gt/+^ mice (CD1 strain) were bred in order to obtain *Ambra1* ^gt/gt^ embryos [11]. Data were obtained in E13.5 embryos by comparing *Ambra1* ^gt/gt^ and wild-type (WT) animals. Mice were housed in individual cages in an environmentally controlled room (23 °C, 12 h light/12 h dark cycle) and provided with food and water *ad libitum*. All animal procedures were approved by the Ethics Committee of the University of Padova and Tor Vergata, Rome.

### MO microinjection

MO (Gene Tools) treatment was performed with MOs against the ATG translation initiation sites of either *ambra1a* or *ambra1b* transcripts (MO-*ambra1a*-ATG and MO-*ambra1b*-ATG) and with splice-blocking MOs designed at the exon 3-intron 3 junction sequence of both genes (MO-*ambra1a*-splice and MO-*ambra1b*-splice). The designed splice-blocking MOs cause the skipping of exon 3, thus altering the translation reading frame of exon 4 with introduction of a premature stop codon, and the resulting proteins lack all known binding domains (Fig. S1). As controls, we used five-nucleotide-mismatched control MOs (MO-*ambra1a*-5m and MO-*ambra1b*-5m). All MOs were previously described and validated [14], however lower MOs dosages were used in this work in order to reduce embryo mortality. Specifically, for each MO, 10.3 ng were injected in the yolk of 1-cell stage embryos, whereas the dosage was halved in the co-injection experiments. Injections were performed under a dissecting microscope using a microinjector attached to a micromanipulator (Leica Microsystems). MOs-injected embryos were then incubated in 1x fish water solution at 28.5 °C up to the desired stages of development.

### RNA synthesis and injections

For the *ambra1*-MO rescue experiments, human *AMBRA1* cDNA was removed from pLPCX-AMBRA1 [11] and subcloned in the pCS2+ vector. Full-length RNA was transcribed using the T3 promoter and the mMessage Machine kit (Ambion) according to the manufacturer's instructions and after plasmid linearization with *Hind*III restriction enzyme. After preliminary experiments with different dosages from 40 to 10 ng/embryo, the 10 ng/embryo dosage was selected for the injection of human *AMBRA1* RNA in one-cell stage embryos for rescue experiments.

### Birefringence assay

Muscle birefringence was analysed by placing anesthetized embryos on a glass polarizing filter and covering with a second polarizing filter on a Leica DMR microscope. Embryos were photographed with a Leica DC500 digital camera. The top filter was twisted until it was possible to see the light refracting through the striated muscle. Pixel intensity in the trunk region was measured with ImageJ software. Values were expressed as the percentage to WT pixel intensity ± SEM (*n* = 20).

### Whole mount in situ hybridization (WMISH)

Zebrafish embryos were fixed overnight in 4% paraformaldehyde (PFA, Sigma) in phosphate-buffered saline (PBS) at the required stages of development. WMISH was performed as previously described [19]. DIG-labeled *myoD1* riboprobe was synthesized by *in vitro* transcription with T7 RNA polymerases (Roche), following the manufacturer's instructions and after plasmid linearization with *Bam*HI restriction enzyme.

### Imaging

For confocal microscopy, fixed embryos were embedded in 0.8% low-melting agarose and placed on a depression slide, and a Nikon C2 confocal system was used to record images. WMISH-stained embryos were mounted in 87% glycerol in PBT (PBS plus 0.1% Tween 20) or cleared and mounted in 2∶1 benzyl benzoate/benzyl alcohol, observed under a Leica DMR microscope, and photographed with a Leica DC500 digital camera.

### Histology and immunofluorescence

Zebrafish embryos were fixed overnight in 4% paraformaldehyde in PBS at 4 °C. For histology, 5 µm thin paraffin sections were cut and stained with haematoxylin and eosin. Embryos were fixed for antibody staining with 4% PFA and whole-mount immunohistochemistry was performed according to Dolez et al. [20], using the following primary antibodies: rabbit polyclonal anti-PH3 (1∶1000; Millipore); mouse monoclonal anti-Pax7 (1∶20; Hybridoma Bank), mouse monoclonal anti-F59 (1∶100; Hybridoma Bank); mouse monoclonal anti-F310 (1∶100, Hybridoma Bank); rabbit polyclonal anti-Laminin (1∶400; Sigma). The following secondary antibodies were used: Alexa Fluor 488 goat anti-mouse IgG1(y1) (A-21121, Invitrogen); Alexa Fluor 594 goat anti-rabbit IgG (H+L) (A-11012, Invitrogen). E13.5 mouse embryos were fixed in 4% paraformaldehyde, de-hydrated and included in paraffin. Haematoxylin-eosin staining was performed following standard protocols. Images were detected using a Zeiss Axioplan microscope equipped with a Leica DC500 digital camera.

Phospho-H3 immunostaining was quantified by counting positive cells present in the same six somites of the trunk region in ten embryos of each category.

### Transmission electron microscopy

Samples were fixed in 6% glutaraldehyde in 0.1 M cacodylate buffer (pH 6.9) overnight at 4°C. After washing in cacodylate buffer, the specimens were post-fixed in 1% OsO_4_ in the same buffer for 2 h and dehydrated in a graded ethanol series followed by propylene oxide. The specimens were embedded in EPON 812 resin. Thick sections (1 µm) were cut with an Ultracut S Reichert ultramicrotome, counterstained with toluidine blue and examined with a light microscope. Thin sections (100 nm) were stained with uranyl acetate and lead citrate. Micrographs were taken with a FEI Tecnai G12 electron microscope operating at 100 kV.

### Microinjection of the hsp70l:Lc3-RFP plasmid into fertilized eggs

A total of 25 ng/µl of hsp70l:Lc3-RFP [21] plasmid was co-injected with each ambra1-MO into zebrafish embryos at one-cell stage. Microinjected embryos were raised to 3 dpf stage and heat shocked by replacing the embryo medium with fish water preheated at 41 °C and then incubated in an air incubator at 38 °C for 30 min to induce hsp70 expression. Lc3-RFP labeled puncta were analysed by confocal microscopy.

### Statistical analysis

Statistical analysis was performed by one-way analysis of variance (ANOVA) followed by Bonferroni's multiple comparison test (GraphPad Prism Software).

## Results

### Knockdown of *ambra1a* and *ambra1b* interferes with embryo motility and muscle integrity

**Figure 1:**
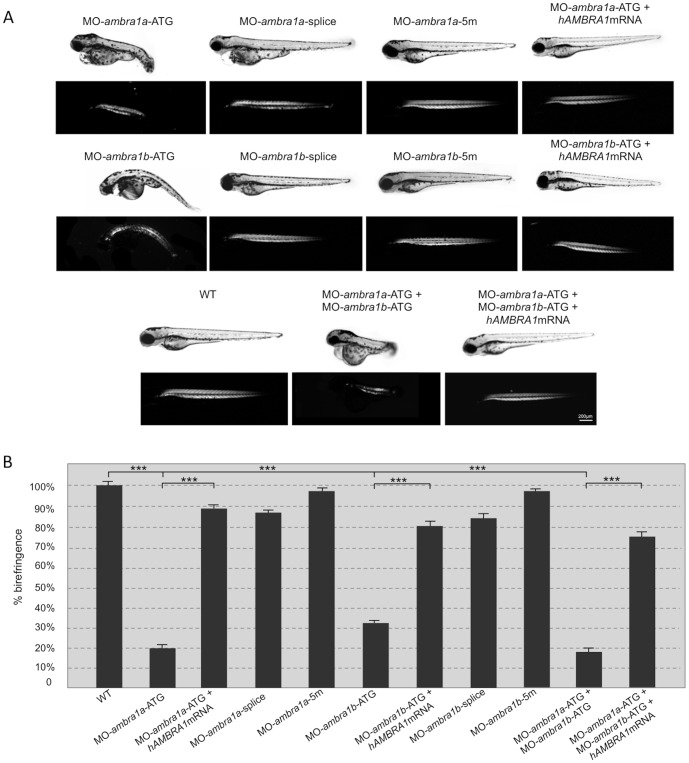
Ablation of *ambra1* results in reduced birefringence in zebrafish embryos. (**A**). Representative images under normal and polarized light of 3-dpf live embryos injected with the indicated MOs. ATG-morphant embryos show reduced size, curved shape, pericardial edema and reduced birefringence when compared to WT and 5 m-control embryos. No visible abnormalities are evident in splice-morphants. The phenotypic defects of ATG-morphant embryos, including birefringence, are rescued by co-injection with 10 ng/embryo of human *AMBRA1* mRNA. (**B**). Quantification of embryo trunk muscles birefringence shows a severe and statistically significant reduction in *ambra1* ATG- and in co-injected morphants. The birefringence is faintly reduced in *ambra1* splice-morphants, whereas WT and 5 m-control embryos display highly birefringent skeletal muscles. Muscle birefringence is statistically increased when ATG- and co-injected morphants are co-injected with human *AMBRA1* mRNA (***, *P*<0.001).

To investigate the effects of *ambra1a* and *ambra1b* ablation during muscle development, we injected validated antisense MOs [14] into the yolk mass of 1-cell embryos to suppress translation of both maternal and zygotic mRNA (ATG MOs) or to silence zygotic transcription of the two genes (splice-blocking MOs). In agreement with a previous work [14], ATG-morphant embryos displayed severe abnormalities in their overall appearance that mainly consisted in body growth delay, curved shape, hemorrhagic pericardial cavity, as well as neural tube defects. The percentages of normal, abnormal and dead embryos at 3 dpf are reported on Fig. S2. More than 70% of ATG-morphant and about 35% of splice-morphant embryos had to be manually dechorionated (Fig. S3). The delay in hatching could be due to an overall developmental delay [14], but also to a reduction of the muscle activity that contributes to the exit of the embryos from their protective outer chorion. Moreover, after hatching, both *ambra1a* and *ambra1b* ATG-morphants, as well as co-injected morphant embryos, showed impaired or totally absent locomotor activity and did not respond to touch with the escape response normally observed in control embryos injected with mismatch MOs (Fig. S4). Less severe aspects of the phenotypes included uncoordinated movements in response to tactile stimuli and often swimming in a circular fashion. In agreement with the impaired locomotor activity, ATG-morphants exhibited a marked and statistically significant reduction of birefringence of the skeletal musculature when compared with control embryos, indicating decreased striated muscle formation and/or loss of myofiber organization (Fig. 1). Reduction of birefringence was comparable between co-injected and *ambra1a* ATG-morphants, and less marked in *ambra1b* ATG-morphants. Birefringence was also slightly reduced in both *ambra1a* and *ambra1b* splice-morphants, whereas it was normal in 5m-control embryos (Fig. 1). Co-injection of ATG-MOs with human *AMBRA1* mRNA resulted in the rescue of the morphants phenotypes (Fig. 1). Moreover, analysis of rescued embryos showed a marked and statistically significant improvement of skeletal muscle birefringence when compared to ATG-morphants (Fig. 1) as well as percentage of hatched embryos at 3 dpf (Fig. S3).

### *ambra1* depletion interferes with *myoD1* expression during myogenesis

**Figure 2:**
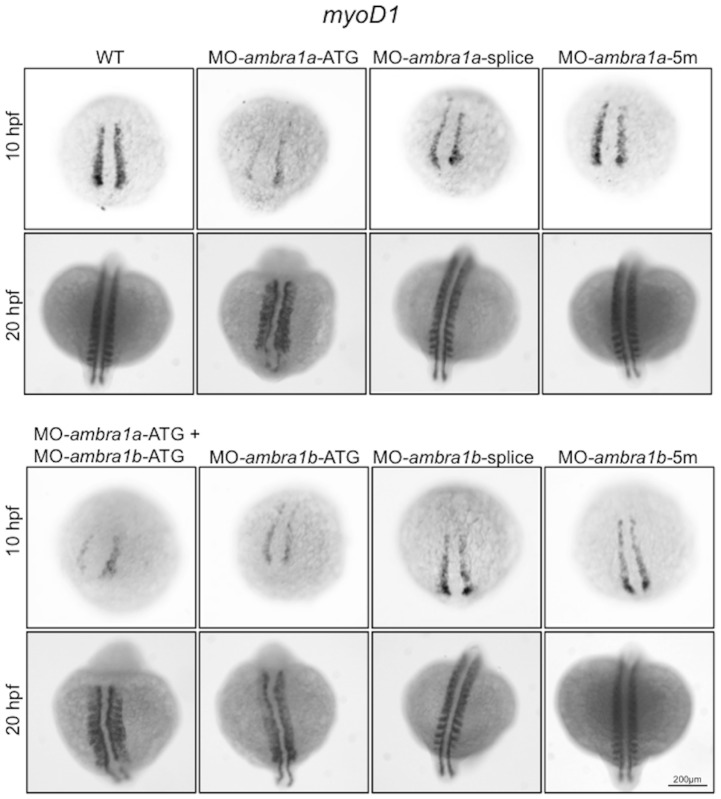
*In situ* hybridization analysis of *myoD1* expression in *ambra1* knockdown embryos. Expression of *myoD1*, analyzed in embryos injected with the indicated MOs at 10 and 20 hpf, is affected in *ambra1* ATG-morphants and in co-injected morphants. No differences are evident in *ambra1* splice-morphants when compared to WT and 5 m-control embryos. Embryos are shown by dorsal view, anterior side on the top.

To assess the requirement for *ambra1a* and *ambra1b* during embryonic myogenesis, we performed WMISH analysis with the somite-specific marker *myoD1*. At the bud stage (10 hpf), expression of *myoD1* in the adaxial cells was reduced in ATG-morphants, while the width between them was increased, particularly at the posterior end (Fig. 2). This was more evident in *ambra1a* ATG-morphants and in co-injected morphants. Expression of *myoD1* appeared normal in splice-morphants for both genes as well as in 5m-control morphants. At 20 hpf, ambra1 ablation resulted in abnormally shaped and less distinct somites, suggesting impaired somite organization, together with shortened anterior/posterior axes and undulated notochord. All defects were more evident in *ambra1a* ATG-morphants and in co-injected embryos. Segmentation pattern appeared normal in splice-morphants and in 5m-control morphants (Fig. 2).

### *ambra1* deficiency leads to abnormal myogenesis

**Figure 3:**
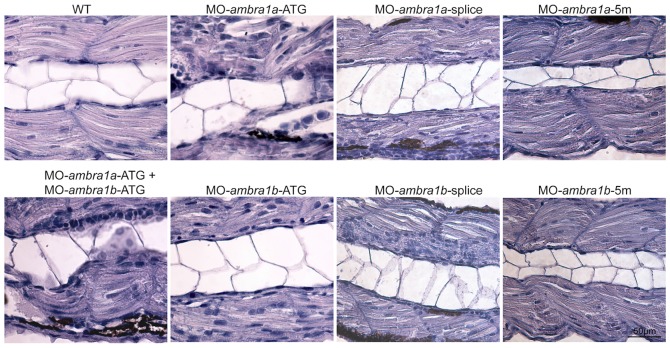
Abnormal morphology of *ambra1* knockdown embryos, as revealed by haematoxylin/eosin staining. Representative longitudinal sections of 3 dpf control and *ambra1* morphant embryos. Myofibers of ATG- and co-injected morphants muscles are highly disorganized and display irregular myosepta boundaries. The phenotype of splice-morphants is much less severe when compared to WT and 5 m-control embryos.

To fully appreciate at a microscopic level the phenotype of *ambra1* morphants, we performed a histological analysis of haematoxylin/eosin stained longitudinal sections of muscles from 3 dpf embryos. This showed well-organized myofibers with elongated nuclei in WT and 5m-control muscles. In ATG-morphants, myosepta were not clearly evident, particularly in *ambra1a* ATG-morphants and co-injected embryos, and myofibers appeared misaligned with markedly disorganized shape and orientation. Moreover, ATG-morphants displayed an apparently increased number of myonuclei (Fig. 3). Morphological changes were much less evident in splice-morphants, although the myosepta were thinner and myofibers appeared less organized with respect to controls.

**Figure 4:**
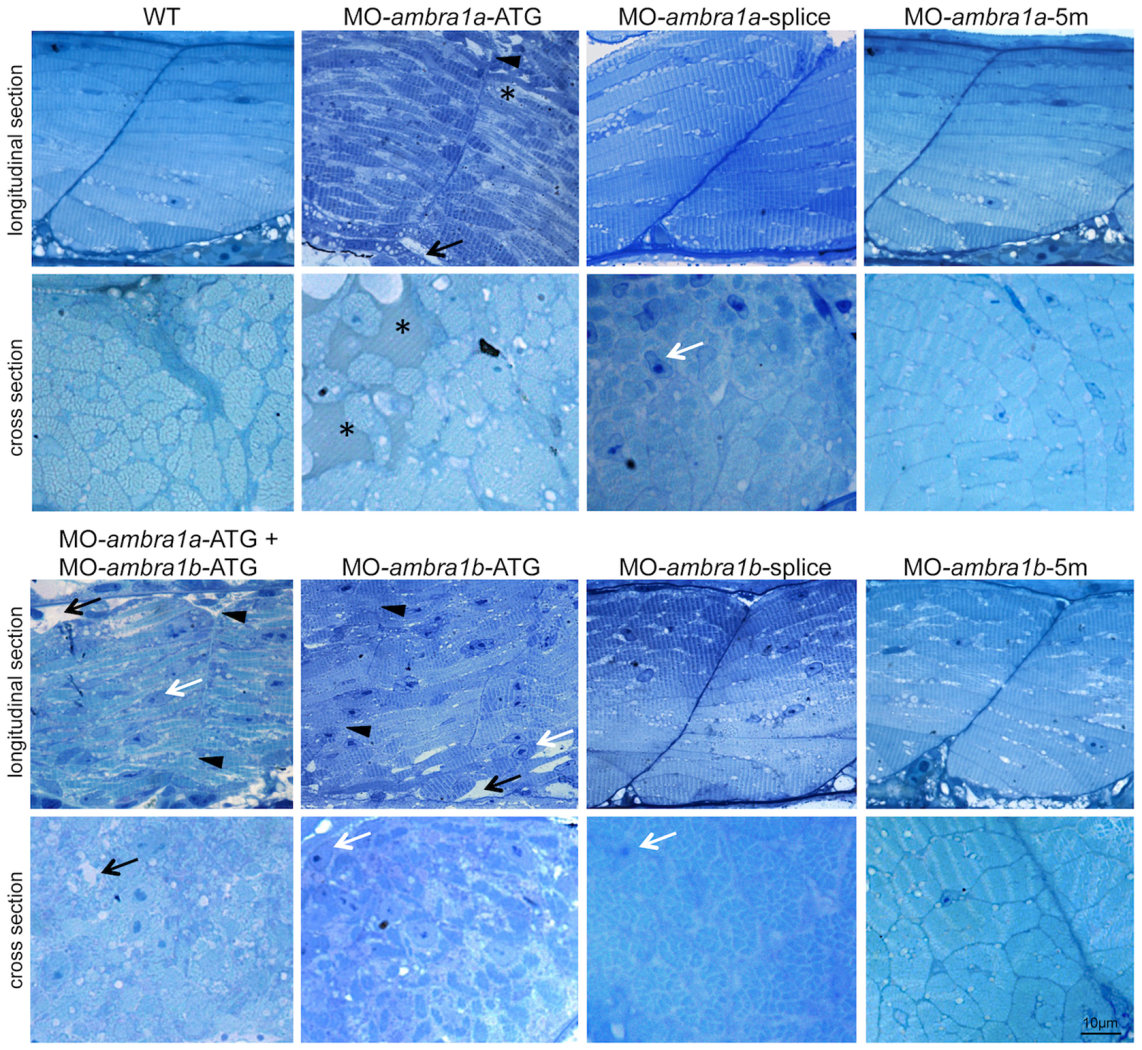
Abnormal morphology of *ambra1* knockdown embryos, as revealed by toluidine blue staining. Representative longitudinal and cross sections of 3 dpf control and *ambra1* morphant embryos. Muscles of *ambra1* ATG-morphants show a severe phenotype, with misaligned myofibers scattered in the somitic compartment. Black arrows, areas devoid of staining; white arrows, large myonuclei with condensed chromatin; black arrowheads, interruption of myoseptum; asterisks, opaque material replacing lost myofibers.

The irregular arrangement of muscle fibers was confirmed by toluidine blue stained semithin longitudinal sections of muscle fibers running between the vertical myosepta (Fig. 4). In both *ambra1a* and *ambra1b* ATG-morphants, the myoseptum was difficult to distinguish and in some places it appeared interrupted. Multiple areas devoid of staining were present within myofibers of both ATG-morphants, and amorphous opaque material replaced lost myofibers. In *ambra1a* ATG-morphants and in co-injected morphants many fibers appeared detached. Skeletal muscles of splice-morphants displayed only minor modifications. Cross sections of the trunk region analysed by toluidine blue staining showed extensive disruption of myofiber structure and organization, with empty spaces and regions filled with amorphous opaque material. Several myonuclei appeared large, abnormally rounded, and centrally localized (Fig. 4).

**Figure 5:**
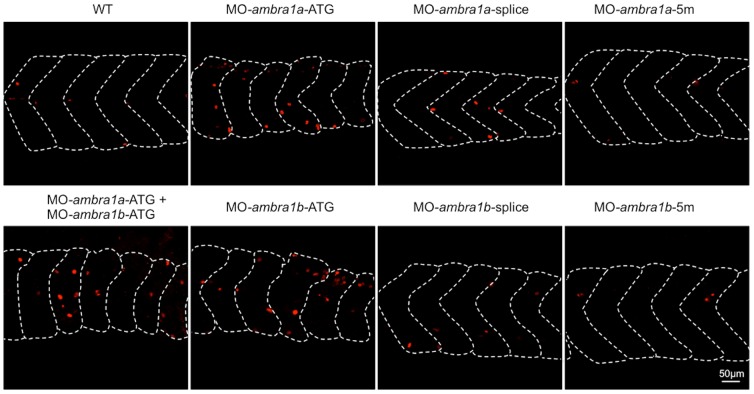
Cell proliferation in muscles of 3 dpf control and *ambra1* morphant embryos. Mitotic cells, detected by immunostaining for phospho-histone H3 in longitudinal sections, are more abundant in ATG-morphant embryos with respect to WT and 5 m-control embryos. Anterior is to the left and dorsal up.

Immunostaining of 3 dpf embryos for phospho-histone H3, a mitotic marker, showed a higher proliferation rate in ATG-morphant embryos (Fig. 5), while the spots of phospho-histone H3 positive nuclei in splice-morphants were only weakly increased compared to controls. The increase of mitotic cells in ATG-morphant embryos resulted statistically significant (Fig. S5).

To evaluate the effect of zebrafish *ambra1a* and *ambra1b* knockdown on the autophagic process, we analysed autophagy in muscle fibers where transient expression of the lysosomal *Lc3*-RFP reporter protein was obtained by microinjection of zebrafish embryos with hsp70l:Lc3-RFP reporter construct, in which the zebrafish *Lc3* gene is driven by the hsp70l promoter and thus induced by heat-shock treatment [21]. Analysis at 3 dpf showed that several Lc3-RFP puncta were present in myofibers from WT and 5 m-control embryos. In contrast, knockdown of either *ambra1a* or *ambra1b* led to an almost complete lack of Lc3-RFP puncta in muscle fibers (Fig. S6).

**Figure 6:**
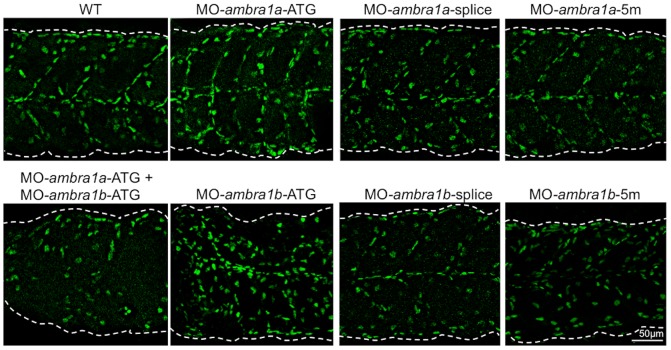
Pax7 expression in control and *ambra1* morphant embryos. Lateral views of 2 dpf muscles analyzed by immunofluorescence for Pax7. In WT and 5-control embryos, Pax7-positive cells are localized at the edge of somites, whereas in *ambra1* morphants many Pax7-positive cells are misplaced in the spaces between myosepta. Anterior is to the left and dorsal up.

Immunofluorescence at 2 dpf for Pax7, a key regulator of muscle progenitor cells [22], showed that both ATG- and splice-morphants had a higher incidence of Pax7-positive cells in the spaces between myosepta, instead of being regularly localized to the edges of the somites as in WT and 5 m-control embryos (Fig. 6).

### Ablation of *ambra1* causes ultrastructural defects of myofibers

**Figure 7:**
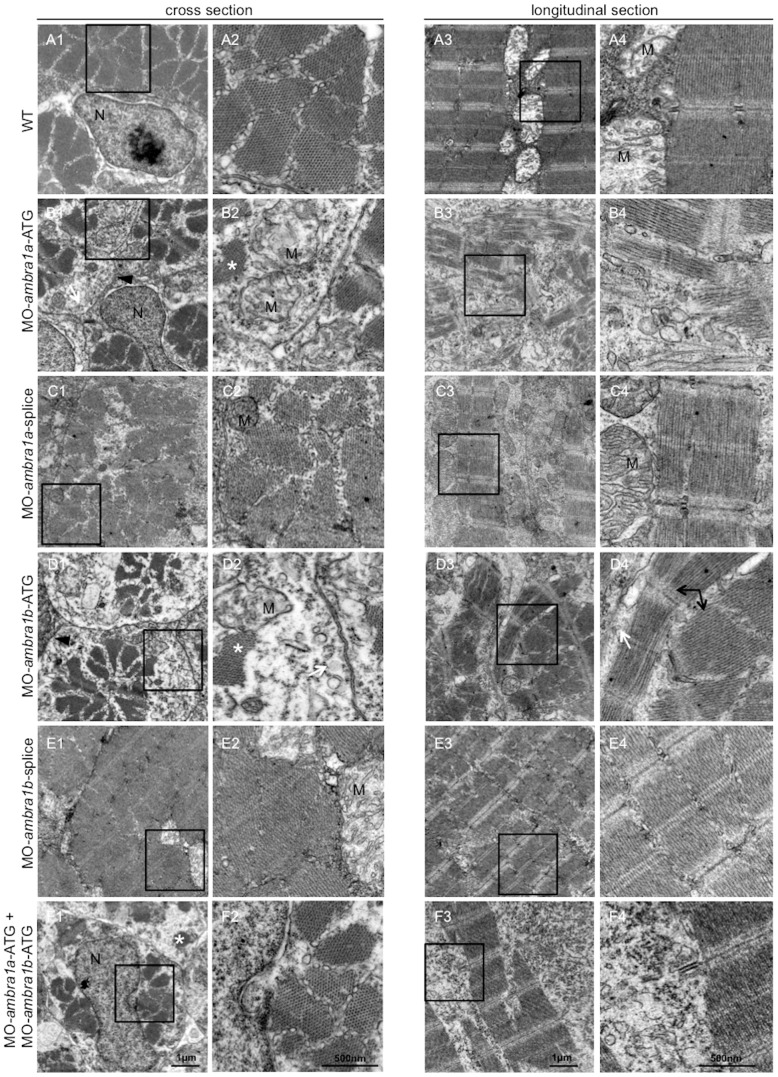
Ultrastructural analysis of *ambra1* morphants muscles reveals disorganized sarcomeres. Representative electron micrographs of cross and longitudinal sections of 3 dpf (WT, panels A1–A4), *ambra1a* ATG-morphant (panels B1-B4), *ambra1a* splice-morphant (panels C1–C4), *ambra1b* ATG-morphant (panels D1–D4), *ambra1b* splice-morphant (panels E1-E4), and co-injected morphant (panels F1–F4) zebrafish embryos. Columns 2 and 4 show higher magnification views of the boxed areas in column 1 and 3, respectively. Muscles of WT and 5 m-control (not shown) embryos display well-organized myofibers, showing densely packed sarcomeres with regular organization of thin and thick myofilaments. *ambra1* depleted muscles show a number of ultrastructural defects, with small patches of disorganized myofibers and mitochondria scattered throughout the cytoplasm. Black arrows, area with myofibrils having different orientations; white arrow, dilated sarcoplasmic reticulum not in contact with myofibrils; asterisks, fragments of torn myofibrils; M, mitochondria; N, nucleus.

To better understand the subcellular alterations responsible for the observed fiber disturbances, we performed an electron microscopy analysis of longitudinal and cross-sections. Zebrafish embryos at 3 dpf showed that WT and 5 m-control embryos (not shown) had well-defined muscle fiber structure with normally developed sarcoplasmic reticulum encircling myofibrils and inter-myofibrillar mitochondria. In control embryos, sarcomeres were clearly visible and formed regular repeating units with alignment of well-defined Z-lines, mitochondria were aligned in rows and had tightly packed cristae, and myofibers were surrounded by very small areas of amorphous material (Fig. 7, panels A1–A4). In contrast, muscle fibers of ATG-morphants and of co-injected embryos contained only small areas of organized filaments, widely dispersed throughout the cells and surrounded by enlarged areas of disorganized cytoplasm devoid of normally appearing organelles. Remnants of degenerating myofibers were also seen in these regions (Fig. 7, panels B1–B4, D1–D4, F1–F4). Although ATG-morphants muscle fibers contained sarcomeres, they were substantially reduced in number, torn, not correctly aligned and dispersed within amorphous material. Areas with myofibrils showing orthogonal arrangement to each other were also visible (Fig. 7). The ultrastructural muscle defects were less severe in splice-morphants, where only small regions devoid of myofibrils were present together with a milder disorganization of sarcomeres (Fig. 7).

**Figure 8:**
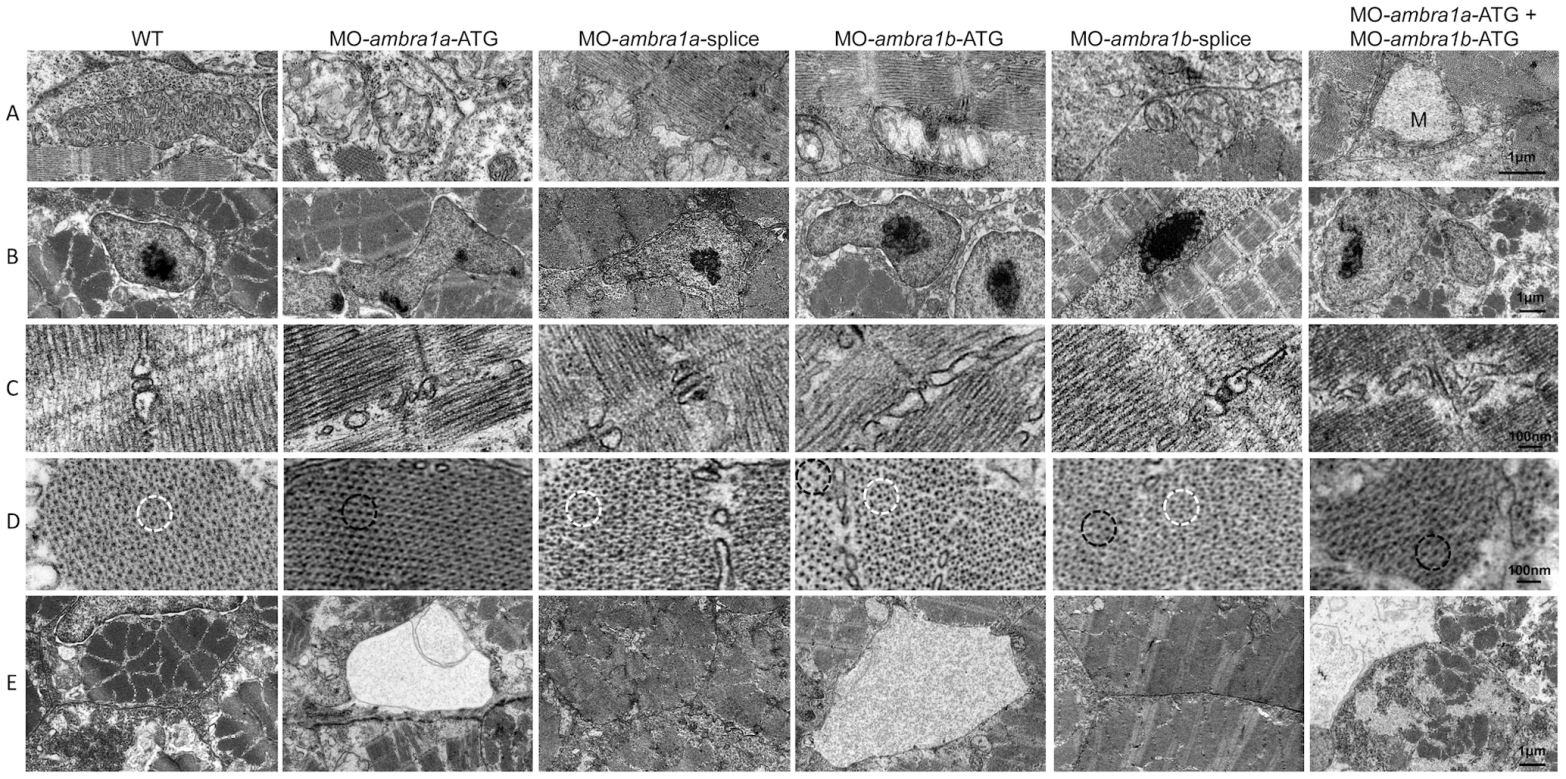
Ultrastructural analysis of *ambra1* morphants muscles reveals disorganized subcellular structures. When compared to WT and 5-control (not shown) embryos, *ambra1* morphant embryos display alterations of mitochondria (row A), nuclei (row B) and triads (row C), perturbation of the hexagonal arrangement of thick and thin filaments (row D), and dilations of the endoplasmic reticulum (row E). Muscles of morphant embryos show the presence of areas with reduced thin filaments (black circles in row D) adjacent to more normal-appearing hexagonal structures (white circles in row D). Co-injected double morphant embryos show exacerbated defects of these structures, whereas defects are barely evident in splice-morphants.

Patterning of internal membranes was also affected in ATG-morphants and in co-injected embryos, as the sarcoplasmic reticulum appeared dilated and often not closely associated with myofibrils (Fig. 8, row E). *ambra1* morphant embryos showed several ultrastructural abnormalities of T-tubules and sarcoplasmic reticulum, ranging from mild changes in splice-morphants to unrecognizable triad areas in co-injected morphants, whereas WT embryos and 5 m-control embryos displayed a normal pattern of T-tubules and sarcoplasmic reticulum resulting in regularly spaced triads (Fig. 8, row C). In *ambra1* morphant embryos, mitochondria were scattered throughout the cytoplasm and their morphology was also markedly affected, as they were often swollen and devoid of cristae or, when present, these were disorganized or abnormal (Fig. 7, panel B2 and Fig. 8, row A). Ultrastructural analysis also confirmed that myonuclei were often larger in morphant embryos, with an irregular shape and more numerous when compared to control embryos (Fig. 8, row B). The abnormal myofiber ultrastructure of ambra1 depleted embryos was particularly evident in cross sections, where myofibers of control embryos showed a regular hexagonal arrays of thick and thin filaments while in *ambra1* morphants the hexagonal arrays were irregular, with areas in which thick filaments were not associated with thin filaments (Fig. 8, row D).

Taken together, these data highlight a severe disorganization of muscle tissue and cells upon ambra1 depletion. Also, alteration of mitochondria and ER structure seem to be causative of the phenotype.

### Ablation of *ambra1* affects both myofibers and myosepta

**Figure 9:**
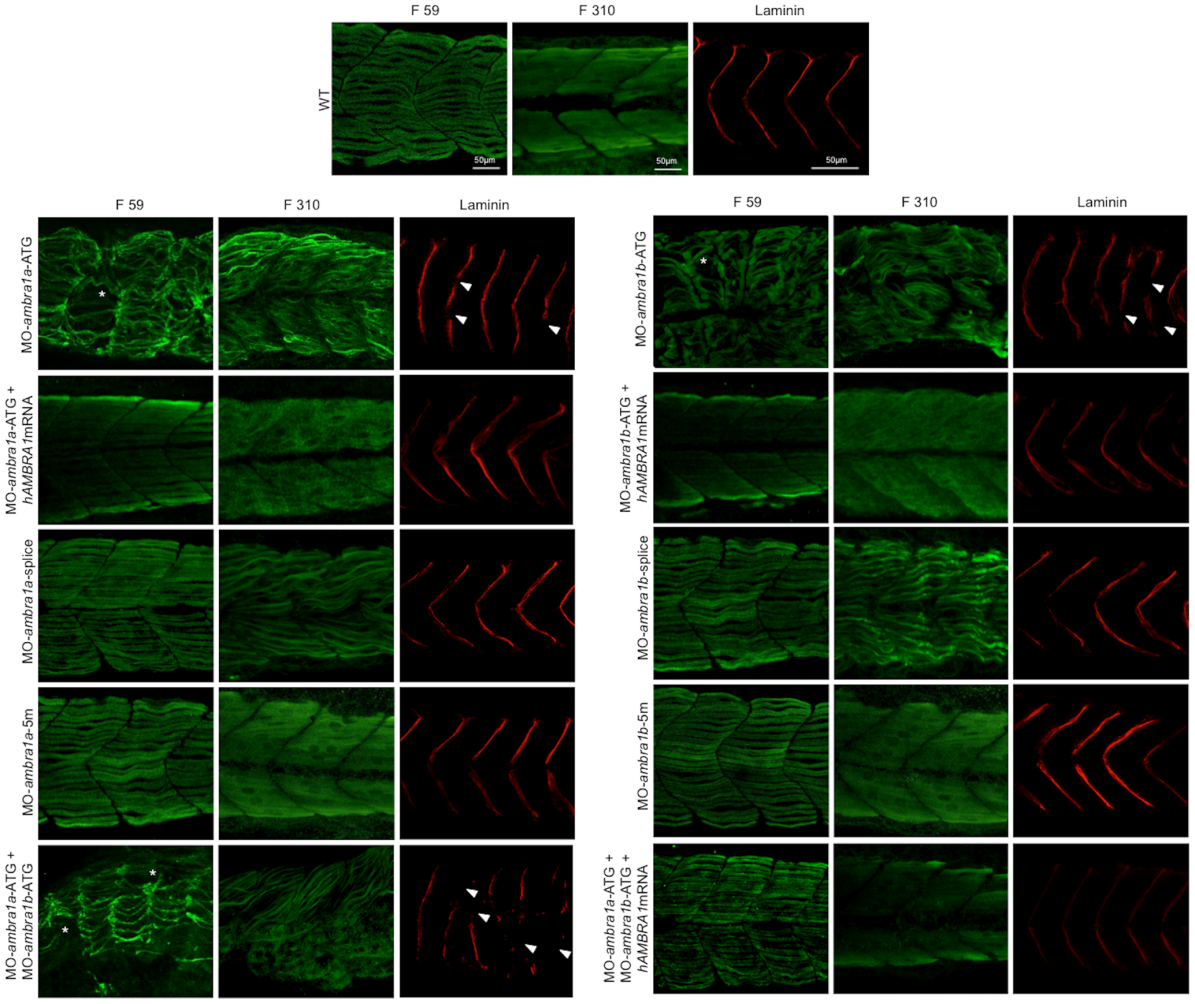
Knockdown of *ambra1* interferes with myosin organization in slow and fast muscles and with myosepta. Lateral views of 3 dpf embryos labeled with the F59 antibody (slow muscle fibers) and with the F310 antibody (fast muscle fibers), showing abnormally organized myofilaments in ATG-morphant embryos when compared to WT and 5 m-control embryos. Slow fibers are thinner in ATG-morphant embryos, whereas fast fibers of splice-morphant embryos display a wavy phenotype, visibly different from controls. The asterisks indicate broken or missing muscle fibers. Laminin labeling highlights the loss of the V-shape arrangement of somites and reveals interrupted myosepta (arrowheads) in ATG-morphant embryos. Defects in myosin organization and myosepta structure are rescued with the by co-injection with human *AMBRA1* mRNA. Anterior is to the left and dorsal up.

Next, to assess whether knockdown of *ambra1a* and *ambra1b* expression results in defective specification and patterning of slow and/or fast muscle fibers, we examined myosin thick filaments in *ambra1* knockdown embryos by immunostaining with the F59 and F310 antibodies, which label slow and fast myosin isoforms, respectively. Slow muscle fibers were still present after *ambra1* knockdown, although myofiber density appeared lower in *ambra1a* ATG-morphants and in co-injected embryos (Fig. 9). However, whereas in control embryos the thick filaments were nicely organized and the myotomal segments were V-shaped and regularly spaced, thick filaments in slow muscles of ATG-morphant embryos appeared highly disorganized, with wavy and twisted myofibrils. Moreover, the characteristic V-shaped appearance of the vertical myoseptum was almost completely absent. Some muscle fibers were missing or detached from the myosepta, generating cell-free spaces in ATG-morphants. Combined injection of both ATG-MOs exacerbated the phenotype. The phenotype was almost normal in *ambra1* splice-morphants, where myofibrils presented only a slightly wavy morphology (Fig. 9). A similar disruption in myofiber organization was also evident in fast muscles. In WT and 5 m-control embryos, the fast muscle fibers appeared relatively uniform in size and were regularly arranged in parallel rows. Conversely, in ATG-morphants fast muscle fibers displayed variable shapes and were highly disorganized, showing a dystrophic appearance with detachment and retraction of myofibers from the vertical myosepta forming the somite boundaries and with irregular and wavy myofibers morphology (Fig. 9). Interestingly, splice-morphants displayed a more evident phenotype in fast muscle fibers than in slow muscle fibers. These data indicated that although slow and fast muscle fibers were still present and in the correct positions within the somites, myofiber organization was disrupted.

To further evaluate the myosepta defects suggested by the morphological analysis, we performed whole-mount immunofluorescence with an anti-Laminin antibody. Myotomes of ATG- and co-injected morphants embryos displayed an abnormal U-shape appearance, with myosepta presenting frequent interruptions of the structure, whereas myosepta appeared normal in control-MOs, as well as in splice-MOs injected embryos (Fig. 9).

Defects on both myofibers and myosepta morphology and organization were rescued by co-injection with human *AMBRA1* mRNA (Fig. 9).

### Skeletal muscles in *Ambra1* ^gt/gt^ mouse embryos display morphological defects

**Figure 10:**
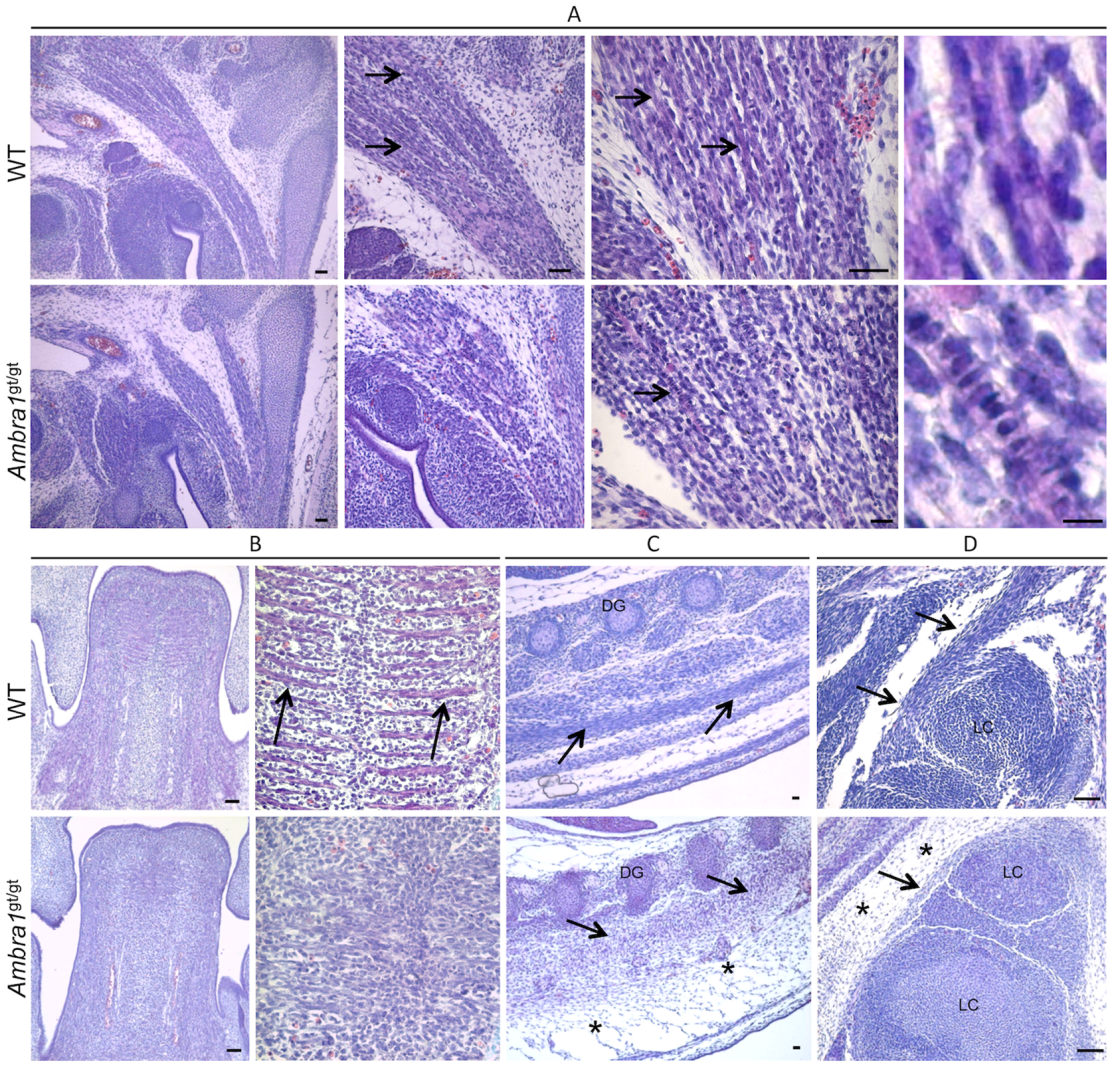
Morphological alteration of skeletal muscles in *Ambra^gt/gt^* mouse embryos. Representative pictures of skeletal muscles from WT and *Ambra1* ^gt/gt^ E13.5 mouse embryos, following haematoxylin-eosin staining. (**A**) Details of neck muscle. WT embryos display several well-organized and mature myofibers (black arrows), which have myonuclei already localized at the edge of the cell (right panel). In *Ambra1* ^gt/gt^ embryos, the muscle is much more immature, with poorly organized myofibers displaying centrally located nuclei (black arrows). (**B**) Details of the tongue. In WT embryos, myofiber are formed and well-organized (arrows), whereas in *Ambra1* ^gt/gt^ embryos there is a general disorganization of muscle architecture. (**C**, **D**) Representative pictures of dorsal (C) and limb (D) muscles (black arrows). *Ambra1* ^gt/gt^ embryos show abnormal muscle organization, together with a marked thickening of the connective tissue (black asterisks). All the analyzed muscles also display a noticeable increase of cell density in *Ambra1* ^gt/gt^ embryos. Scale bar, 50 µm.

To obtain further insight into the role of Ambra1 during muscle development, we investigated skeletal muscles from *Ambra1* ^gt/gt^ mutant mice, which bear a gene trap insertion in the *Ambra1* gene [11]. We first analyzed muscle morphology by haematoxylin-eosin staining in E13.5 mouse embryos, when differentiation of muscles is not yet complete and myogenic cells are undergoing fusion to form myofibers. In WT E13.5 mouse embryos, the structure of the developing muscles appeared normal, and myofibers were well organized and aligned to generate the ordered structure of the muscle, with many nuclei already localized at the periphery (Fig. 10A, upper panels). In *Ambra1* ^gt/gt^ E13.5 mouse embryos, skeletal muscles were formed but the three-dimensional tissue architecture was less organized (Fig. 10A, lower panels). The abnormal structure of developing muscles in *Ambra1* ^gt/gt^ embryos may be due to a failure in completing muscle development or to a delay in myofiber maturation. This was also supported by the presence of many immature myofibers displaying centrally located nuclei in *Ambra1* ^gt/gt^ embryos (Fig. 10A, lower panels). These features, which were present in all muscles of *Ambra1* ^gt/gt^ embryos we analyzed, are clearly exemplified by the developing tongue. In WT E13.5 embryos, tongue showed a well-defined array of parallel myofibers distinctly organized in the three different planes of the developing intrinsic tongue muscles (Fig. 10B, upper panels). Conversely, in *Ambra1* ^gt/gt^ E13.5 embryos, the tongue displayed a general disorganization of the developing muscles and myofibers were less defined. In addition, there was a marked increase of cell density in the whole muscle tissue of *Ambra1* ^gt/gt^ embryos (Fig. 10B, lower panels). Similar morphological and cellular alterations were present in the developing dorsal and limb muscles of *Ambra1* ^gt/gt^ embryos, which also showed a noticeable increase of the interstitial connective tissue (Fig. 10C, D).

## Discussion

In this work we used targeted protein depletion approaches to investigate the involvement of Ambra1 in muscle development. The severe phenotypes displayed by zebrafish *ambra1a*/*ambra1b* morphants and mouse *Ambra1* ^gt/gt^ embryos suggest a key role for Ambra1 in myogenesis. Our analysis on muscles of *ambra1a* and *ambra1b* zebrafish morphants was mainly focused on 3 dpf embryos, as at this stage muscles are fully developed. Our data indicate that ablation of Ambra1 leads to a severe myopathy with structural and functional defects of skeletal muscles, characterized by a marked reduction of myofiber density, abnormal orientation and decreased alignment of myofibers, disorganization of sarcomeres, alterations of the tubulo-reticular network and abnormal mitochondria morphology. The reduced locomotor activity of *ambra1a*/*ambra1b* zebrafish morphants, as well as the myofiber and myosepta defects, were rescued by co-injection with human *AMBRA1* mRNA, thus confirming that these defects are caused by Ambra1 depletion and pointing at the conservation of Ambra1 functions during evolution. Although a role for Ambra1 in skeletal muscle was never proposed before now, our findings are in agreement with other recent studies indicating that autophagy plays a key role in skeletal muscles, as shown by the myopathic phenotype of *Atg5* and *Atg7* muscle-specific knockout mice [3], [4] and by the connection between autophagy deregulation and muscular dystrophies [5]–[9].

In zebrafish, transcripts for both *ambra1* genes are present as maternal RNAs in the eggs and display a gradual decline until 8 hpf, being replaced by zygotic mRNAs from 12 hpf onwards [14]. To verify a possible role of these proteins in the early stages of skeletal muscle development, the commitment to the myogenic fate was analysed by WMISH for *myoD1*, whose expression normally begins in the adaxial cells prior to somite formation [23], [24]. These cells, after migrating laterally through the somites, form the slow muscle cells [25], whereas those remaining near to the notochord form the muscle pioneer cells [26]. Later, *myoD1* expression takes place in the posterior half of each newly formed somite, giving rise to the fast muscle fibers [25]. Knockdown of zebrafish *ambra1* genes led to marked changes of *myoD1* expression, resulting in a reduced *myoD1* signal and a widening of the space between the bilateral adaxial cells of ATG-morphant embryos. At later stages, somites present a broad-shaped and non-homogeneous *myoD1* expression in ATG-morphant embryos. Interestingly, expression of *shh*, a gene coding for a secreted signalling protein implicated in the commitment of muscle precursors (reviewed in [15], [26]), was also found to be abnormal in *Ambra1* ^gt/gt^ mouse embryos [11], as well as in zebrafish *ambra1* ATG-morphant embryos, resulting in notochord waving [14]. Depletion of Ambra1 proteins in zebrafish did not prevent the specification and differentiation of slow and fast muscle fibers, but clearly interfered with myofibrillogenesis leading to an anomalous pattern of both fiber types. As different levels, along with range and timing, of Shh signalling specify different muscle subtypes [27], [28], the displaced expression of this morphogen could explain the disorganization of the *ambra1*-morphant muscle fibers.

The changes in *myoD1* and *shh* expression, together with the reduced locomotor activity, observed in *ambra1* morphants occur at very early developmental stages, when embryonic cells are likely not yet competent for autophagy, e.g. the biological process in which Ambra1 has a well-established role. Actually, autophagy can be observed in zebrafish embryos starting from 32 hpf [29], and thus relatively late compared with the onset of the muscle developmental defects. Interestingly, *ambra1* depletion seems to interfere with the gene expression program responsible for correct muscle development, as suggested by the displaced expression of *shh* [14] and of *myoD1*, however it is still unclear whether these effects are related to Ambra1 pro-autophagic roles. On the other side, *ambra1* morphant embryos were also found to display reduced autophagy, as measured by immunoblot analysis for lipidated LC3 at 2 dpf [14]. A decreased activity of the autophagic process was also suggested by the lower incidence of puncta in muscle fibers isolated from zebrafish embryos expressing a hsp701:Lc3-RFP reporter plasmid.

Pax7 is a key regulator for myogenic progenitor cells in all vertebrates and Pax7-positive cells are already aligned along the vertical and horizontal myosepta at 2 dpf during zebrafish development [30]. In *ambra1* morphant embryos, the regular arrangement of Pax7-positive cells was disturbed and these also occurred in the spaces between myosepta, thus confirming the disorganization of somites. Although a significant increase of Pax7-positive cells was found in zebrafish with dystrophic phenotypes [30], [31], our analysis did not reveal any obvious difference in the number of these cells between control and *ambra1* morphant embryos, This result seems to contrast with the apparently higher proliferation rate seen in *ambra1* morphants both by haematoxylin/eosin staining and by immunofluorescence for phosphorylated histone H3. However, this apparent discrepancy may be explained by a higher proliferation of fibroblasts, as also suggested by muscles of *Ambra1* ^gt/gt^ mouse embryos showing a conspicuous thickening of the interstitial connective tissue.

As for a number of other genes, the zebrafish ortholog of mammalian *Ambra1* gene is present as two duplicated paralogs coding for functional proteins. Although knockdown of one of these two paralogous genes is sufficient to alter muscle structure, the more severe phenotype of embryos deficient for both *ambra1* paralogs suggests that the two proteins not only work in similar biological process but also play distinct roles, thus justifying the retention of both genes as functional in the zebrafish genome after the fish-specific whole-genome duplication [32]. Notably, knockdown of only zygotic *ambra1a* and *ambra1b* transcripts by means of splice-MOs resulted in less severe muscle developmental defects, indicating the importance of maternally supplied *ambra1* transcripts and of the corresponding proteins in early embryonic development. Interestingly, and at difference from other parameters, splice-morphants showed a remarkably stronger phenotype than ATG-morphants with regard to fast fiber development, a finding that is in agreement with the different timing of slow and fast fiber differentiation [33]. Since fast fibers differentiate after slow fibers, the process in this case could be more affected by the silencing of zygotic transcripts because maternal transcript would be no longer available at that stage.

A number of recent studies indicated that autophagy plays important physiological and pathological roles in mature and fully developed skeletal muscles (reviewed in [34], [35]). Our data, obtained from *ambra1a* and *ambra1b* zebrafish morphant embryos and from *Ambra1* ^gt/gt^ mouse embryos, indicate that this autophagy related-protein plays a critical and evolutionally conserved function during skeletal muscle development. In both animal models, ablation of Ambra1 leads to abnormal muscle morphogenesis. Ambra1 is critical not only for the correct architecture and maturation of myofibers, but it seems also implicated in cell proliferation control. Our data suggest a new function for Ambra1 during muscle biogenesis. While the presence of abnormal organelles is likely associated to the well-established role of Ambra1 in autophagy regulation, the altered morphology and the hypercellularity of the developing skeletal muscles may be due to a different function of Ambra1. Further studies will be needed to fully elucidate the functional roles of Ambra1 in the developing and postnatal skeletal muscles, in order to understand how this protein contributes to muscle homeostasis and identify possible human muscle pathologies linked to Ambra1 mutations.

## Supporting Information

Figure S1 **Schematic diagram showing the exon-intron structure of zebrafish** ***ambra1a*** **(A) and** ***ambra1b*** **(B) genes.** The localization of known domains in the corresponding proteins is also indicated. The partial sequences of the abnormally spliced *ambra1a* and *ambra1b* transcripts, following targeting with the splice-MOs, highlights the loss of exon 3 (arrow) causing a codon frameshift and the introduction of a premature stop codon.
(TIF)

Figure S2 **Percentage of dead, abnormal and normal 3 dpf embryos after injection with the different MOs.**
(TIF)

Figure S3 **Quantification of chorion hatching in 72 hpf embryos after injection with the different MOs.**
(TIF)

Figure S4 **Quantification of touch-evoked response and circular movement at 3 dpf.** Embryos were quantified in three independent microinjections, and the number of embryos for each microinjection experiments was about 80.
(TIF)

Figure S5 **Analysis of the number of mitotic cells present in the same six somites region of 10 embryos of each category.** Data are presented as the mean ±SEM. ***, *P*<0.001.
(TIF)

Figure S6 **Fluorescence detection in muscle fibers of control and** ***ambra1*** **morphant embryos following transfection with a Lc3-RFP reporter construct.** Several fluorescent puncta have been detected in the transfected muscle fibers from WT and 5m-morphant embryos whereas only few puncta were visibile in ATG-morphant embryos.
(TIF)

## References

[1] Levine B, Kroemer G (2008) Autophagy in the pathogenesis of disease. Cell 132: 27–42. 18191218 10.1016/j.cell.2007.12.018 PMC2696814

[2] Mizushima N, Komatsu M (2011) Autophagy: renovation of cells and tissues. Cell 147: 728–741. 22078875 10.1016/j.cell.2011.10.026

[3] Raben NV, Hill L, Shea S, Takikita R, Baum N, et al (2008) Suppression of autophagy in skeletal muscle uncovers the accumulation of ubiquitinated proteins and their potential role in muscle damage in Pompe disease. Hum Mol Genet 17: 3897–3908. 18782848 10.1093/hmg/ddn292 PMC2638578

[4] Masiero E, Agatea L, Mammucari C, Blaauw B, Loro E, et al (2009) Autophagy is required to maintain muscle mass. Cell Metab 10: 507–515. 19945408 10.1016/j.cmet.2009.10.008

[5] Grumati P, Coletto L, Sabatelli P, Cescon M, Angelin A, et al (2010) Autophagy is defective in collagen VI muscular dystrophies, and its reactivation rescues myofiber degeneration. Nat Med 16: 1313–1320. 21037586 10.1038/nm.2247

[6] Carmignac V, Svensson M, Körner Z, Elowsson L, Matsumura C, et al (2011) Autophagy is increased in laminin α2 chain-deficient muscle and its inhibition improves muscle morphology in a mouse model of MDC1A. Hum Mol Genet 20: 4891–4902. 21920942 10.1093/hmg/ddr427

[7] De Palma C, Morisi F, Cheli S, Pambianco S, Cappello V, et al (2012) Autophagy as a new therapeutic target in Duchenne muscular dystrophy. Cell Death Dis 15: e418. 10.1038/cddis.2012.159 PMC3542595 23152054

[8] Choi JC, Muchir A, Wu W, Iwata S, Homma S, et al (2012) Temsirolimus activates autophagy and ameliorates cardiomyopathy caused by lamin A/C gene mutation. Sci Transl Med 4: 144ra102. 10.1126/scitranslmed.3003875 PMC3700376 22837537

[9] Ramos FJ, Chen SC, Garelick MG, Dai DF, Liao CY, et al (2012) Rapamycin reverses elevated mTORC1 signaling in lamin A/C-deficient mice, rescues cardiac and skeletal muscle function, and extends survival. Sci Transl Med 4: 144ra103. 10.1126/scitranslmed.3003802 PMC3613228 22837538

[10] Cullup T, Kho AL, Dionisi-Vici C, Brandmeier B, Smith F, et al (2012) Recessive mutations in EPG5 cause Vici syndrome, a multisystem disorder with defective autophagy. Nat Genet 45: 83–87. 23222957 10.1038/ng.2497 PMC4012842

[11] Fimia GM, Stoykova A, Romagnoli A, Giunta L, Di Bartolomeo S, et al (2007) Ambra1 regulates autophagy and development of the nervous system. Nature 447: 1121–1125. 17589504 10.1038/nature05925

[12] Fimia GM, Corazzari M, Antonioli M, Piacentini M (2013) Ambra1 at the crossroad between autophagy and cell death. Oncogene 32: 3311–3318. 23069654 10.1038/onc.2012.455

[13] Di Bartolomeo S, Corazzari M, Oliverio S, Nazio F, Lisi G, et al (2010) The dynamic interaction of Ambra1 with the dynein motor complex regulates mammalian autophagy. J Cell Biol 191: 155–168. 20921139 10.1083/jcb.201002100 PMC2953445

[14] Benato F, Skobo T, Gioacchini G, Moro I, Ciccosanti F, et al (2013) Ambra1 knockdown in zebrafish leads to incomplete development due to severe defects in organogenesis. Autophagy 9: 476–495. 23348054 10.4161/auto.23278 PMC3627665

[15] Jackson HE, Ingham PW (2013) Control of muscle fibre-type diversity during embryonic development: the zebrafish paradigm. Mech Dev 130: 447–457. 23811405 10.1016/j.mod.2013.06.001

[16] Gibbs EM, Horstick EJ, Dowling JJ (2013) Swimming into prominence: the zebrafish as a valuable tool for studying human myopathies and muscular dystrophies. FEBS J 280: 4187–4197. 23809187 10.1111/febs.12412 PMC4017590

[17] Kimmel CB, Ballard WW, Kimmel SR, Ullmann B, Schilling TF (1995) Stages of embryonic development of the zebrafish. Dev Dyn 203: 253–310. 8589427 10.1002/aja.1002030302

[18] Westerfield M (1995) The Zebrafish Book. A Guide for the Laboratory Use of Zebrafish (Danio rerio), 3rd Edition. Eugene, OR, University of Oregon Press.

[19] Thisse C, Thisse B (2008) High-resolution in situ hybridization to whole-mount zebrafish embryos. Nat Protoc 3: 59–69. 18193022 10.1038/nprot.2007.514

[20] Dolez M, Nicolas JF, Hirsinger E (2011) Laminins, via heparan sulfate proteoglycans, participate in zebrafish myotome morphogenesis by modulating the pattern of Bmp responsiveness. Development 138: 97–106. 21115608 10.1242/dev.053975

[21] Ellis K, Bagwell J, Bagnat M (2013) Notochord vacuoles are lysosome-related organelles that function in axis and spine morphogenesis. J Cell Biol 200: 667–679. 23460678 10.1083/jcb.201212095 PMC3587825

[22] Nord H, Nygård Skalman L, von Hofsten J (2013) Six1 regulates proliferation of Pax7-positive muscle progenitors in zebrafish. J Cell Sci 126: 1868–1880. 23444384 10.1242/jcs.119917

[23] Coutelle O, Blagden CS, Hampson R, Halai C, Rigby PW, et al (2001) Hedgehog signalling is required for maintenance of myf5 and myoD expression and timely terminal differentiation in zebrafish adaxial myogenesis. Dev Biol 236: 136–150. 11456450 10.1006/dbio.2001.0193

[24] Weinberg ES, Allende ML, Kelly CS, Abdelhamid A, Murakami T, et al (1996) Developmental regulation of zebrafish MyoD in wild-type, no tail and spadetail embryos. Development 122: 271–280. 8565839 10.1242/dev.122.1.271

[25] Devoto SH, Melançon E, Eisen JS, Westerfield M (1996) Identification of separate slow and fast muscle precursor cells in vivo, prior to somite formation. Development 122: 3371–3380. 8951054 10.1242/dev.122.11.3371

[26] Ochi H, Westerfield M (2007) Signaling networks that regulate muscle development: lessons from zebrafish. Dev Growth Differ 49: 1–11. 17227340 10.1111/j.1440-169X.2007.00905.x

[27] Du SJ, Devoto SH, Westerfield M, Moon RT (1997) Positive and negative regulation of muscle cell identity by members of the hedgehog and TGF-beta gene families. J Cell Biol 139: 145–156. 9314535 10.1083/jcb.139.1.145 PMC2139815

[28] Wolff C, Roy S, Ingham PW (2003) Multiple muscle cell identities induced by distinct levels and timing of hedgehog activity in zebrafish embryos. Curr Biol 13: 1169–1181. 12867027 10.1016/s0960-9822(03)00461-5

[29] He C, Bartholomew CR, Zhou W, Klionsky DJ (2009) Assaying autophagic activity in transgenic GFP-Lc3 and GFP-Gabarap zebrafish embryos. Autophagy 5: 520–526. 19221467 10.4161/auto.5.4.7768 PMC2754832

[30] Seger C, Hargrave M, Wang X, Chai RJ, Elworthy S, et al (2011) Analysis of Pax7 expressing myogenic cells in zebrafish muscle development, injury, and models of disease. Dev Dyn 240: 2440–2451. 21954137 10.1002/dvdy.22745

[31] Berger J, Berger S, Hall TE, Lieschke GJ, Currie PD (2010) Dystrophin-deficient zebrafish feature aspects of the Duchenne muscular dystrophy pathology. Neuromuscul Disord 20: 826–832. 20850317 10.1016/j.nmd.2010.08.004

[32] Postlethwait J, Amores A, Cresko W, Singer A, Yan YL (2004) Subfunction partitioning, the teleost radiation and the annotation of the human genome. Trends Genet 20: 481–490. 15363902 10.1016/j.tig.2004.08.001

[33] Blagden CS, Currie PD, Ingham PW, Hughes SM (1997) Notochord induction of zebrafish slow muscle mediated by Sonic hedgehog. Genes Dev 11: 2163–2175. 9303533 10.1101/gad.11.17.2163 PMC275397

[34] Sandri M, Coletto L, Grumati P, Bonaldo P (2013) Misregulation of autophagy and protein degradation systems in myopathies and muscular dystrophies. J Cell Sci 126: 5325–5333. 24293330 10.1242/jcs.114041

[35] Vainshtein A, Grumati P, Sandri M, Bonaldo P (2014) Skeletal muscle, autophagy, and physical activity: the ménage a trois of metabolic regulation in health and disease. J Mol Med 92: 127–137. 24271008 10.1007/s00109-013-1096-z

