# Correction: Zebrafish *ambra1a* and *ambra1b* Knockdown Impairs Skeletal Muscle Development

**DOI:** 10.1371/journal.pone.0109480

**Published:** 2014-09-26

**Authors:** The PLOS ONE Staff

There is an error in the Author affiliations. The fifth author Valentina Cianfanelli and eighth author Sabrina Di Bartolomeo are also affiliated with [4] Department of Neuroscience, Istituto di Ricovero e Cura a Carattere Scientifico “Santa Lucia Foundation”, Rome, Italy.
