# Supplementary figures and images for: Zebrafish ambra1a and ambra1b Knockdown Impairs Skeletal Muscle Development
Source: PLoS One. 2014 Jun 12;9(6):e99210. doi: 10.1371/journal.pone.0099210 (PMC4055674; doi:10.1371/journal.pone.0099210)

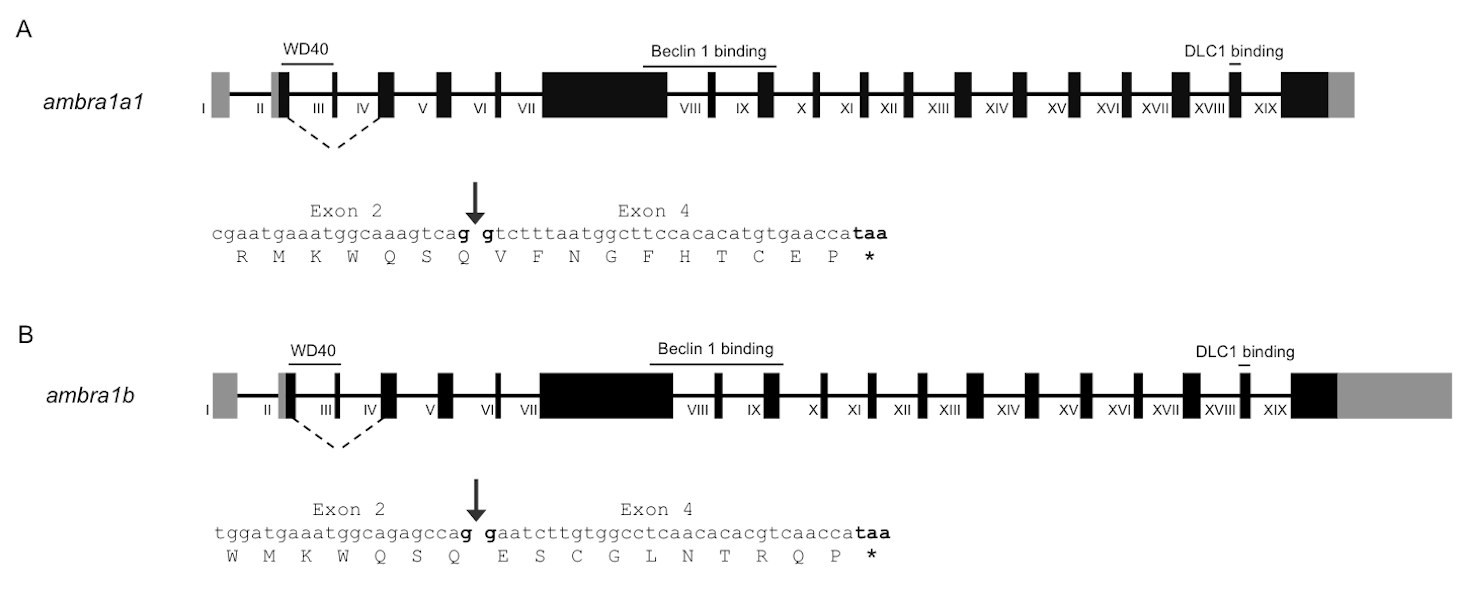

Supplement: Figure S1 — Schematic diagram showing the exon-intron structure of zebrafish ambra1a (A) and ambra1b (B) genes. The localization of known domains in the corresponding proteins is also indicated. The partial sequences of the abnormally spliced ambra1a and ambra1b transcripts, following targeting with the splice-MOs, highlights the loss of exon 3 (arrow) causing a codon frameshift and the introduction of a premature stop codon. (TIF) [file pone.0099210.s001.tif]

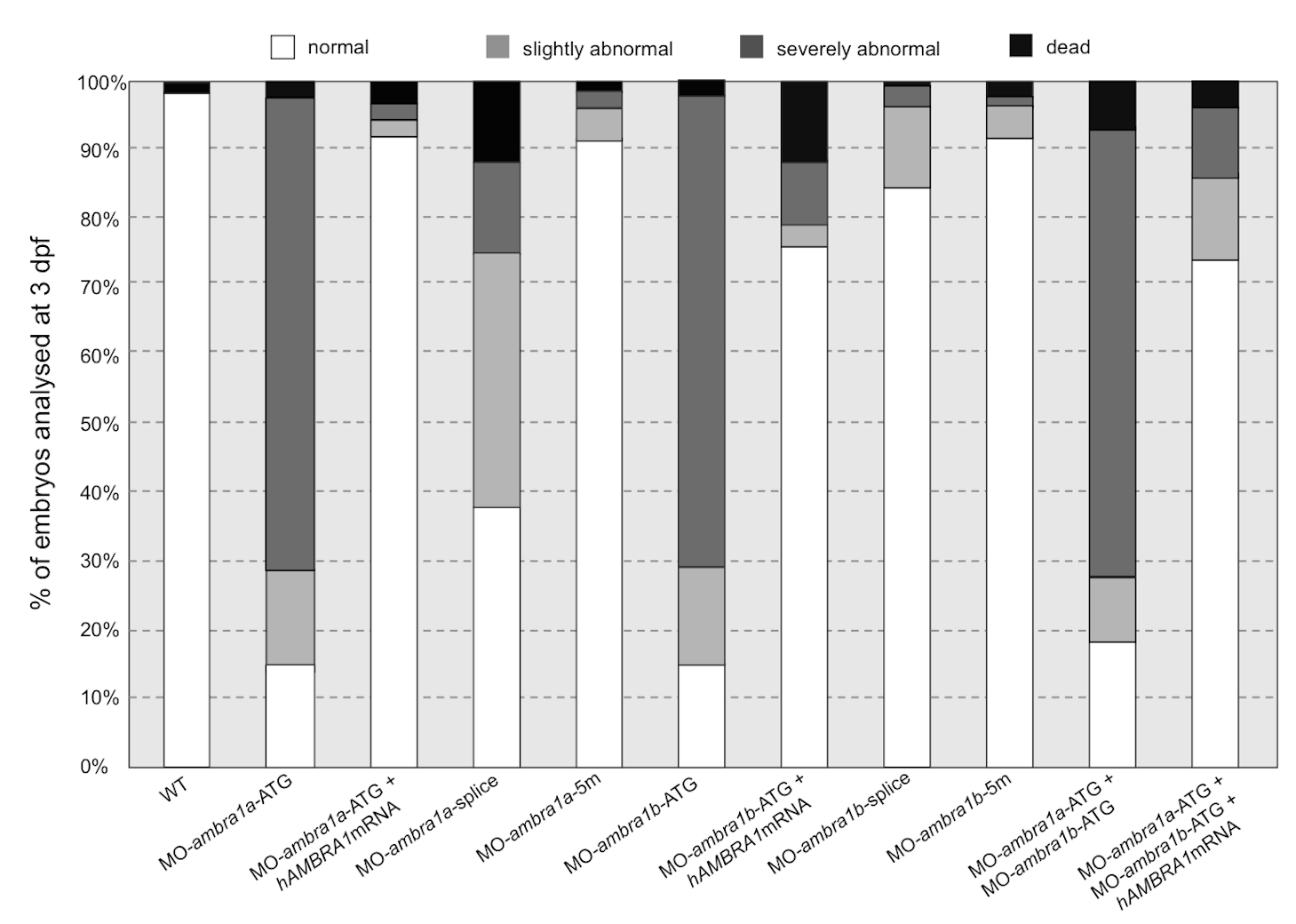

Supplement: Figure S2 — Percentage of dead, abnormal and normal 3 dpf embryos after injection with the different MOs. (TIF) [file pone.0099210.s002.tif]

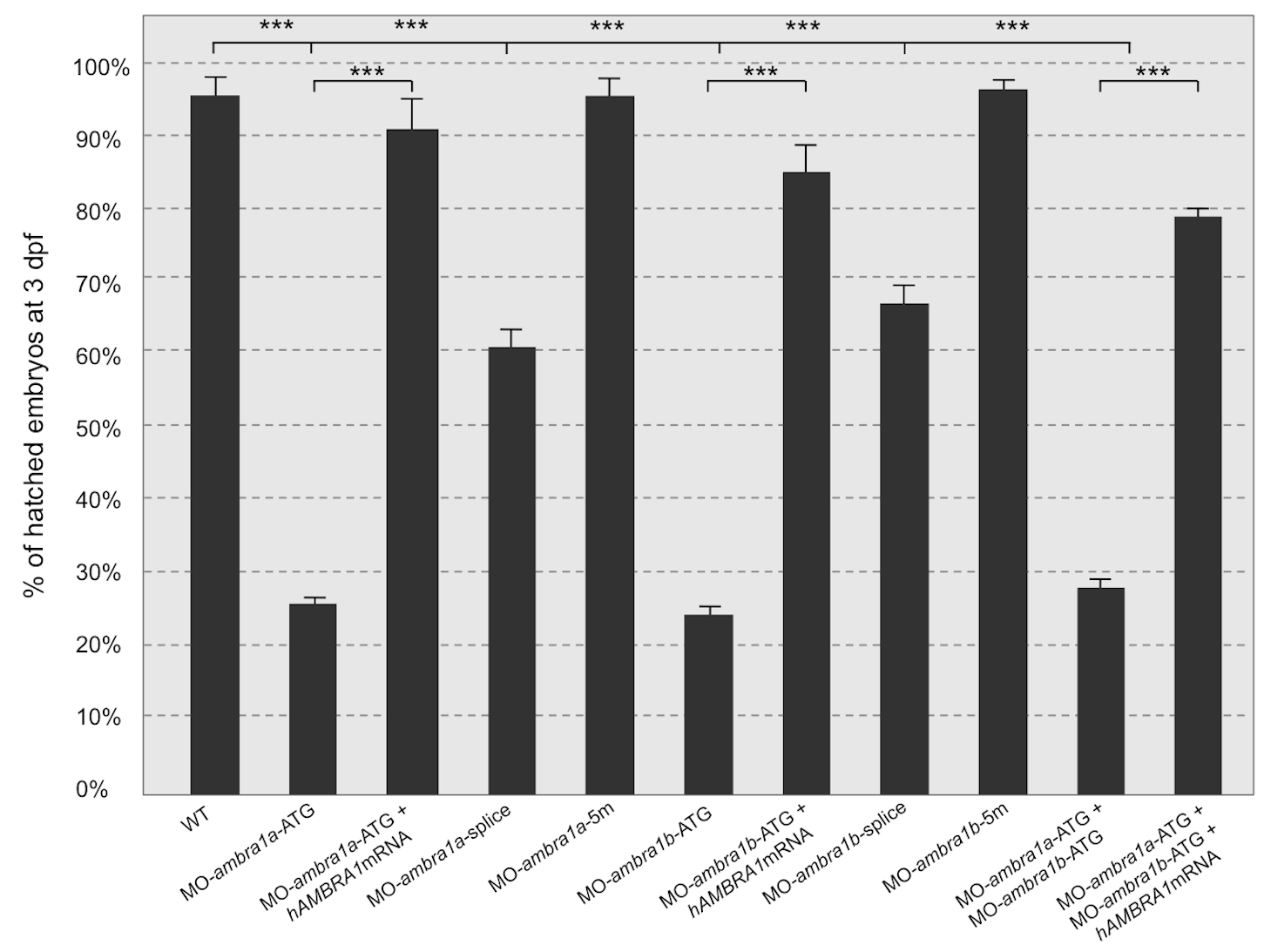

Supplement: Figure S3 — Quantification of chorion hatching in 72 hpf embryos after injection with the different MOs. (TIF) [file pone.0099210.s003.tif]

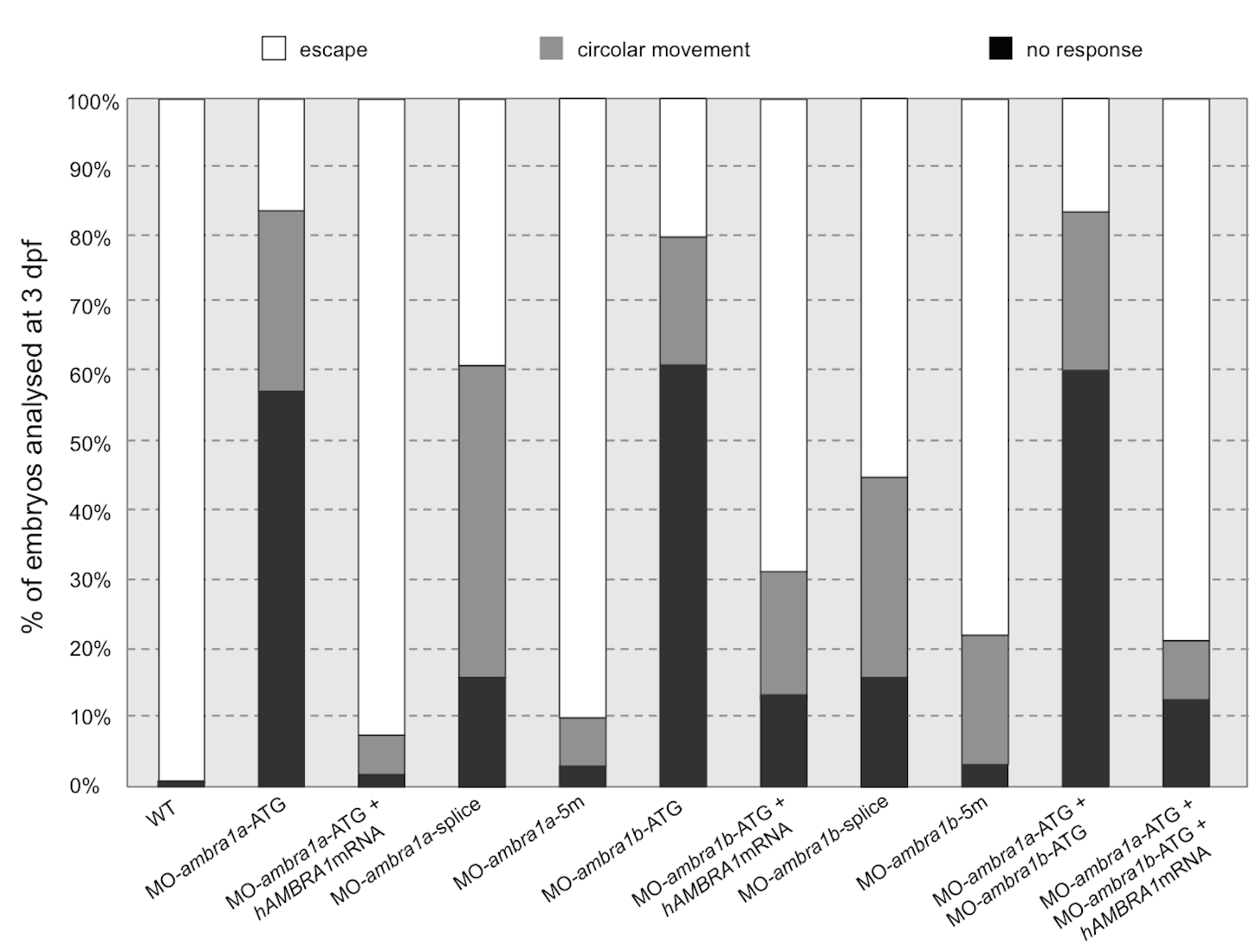

Supplement: Figure S4 — Quantification of touch-evoked response and circular movement at 3 dpf. Embryos were quantified in three independent microinjections, and the number of embryos for each microinjection experiments was about 80. (TIF) [file pone.0099210.s004.tif]

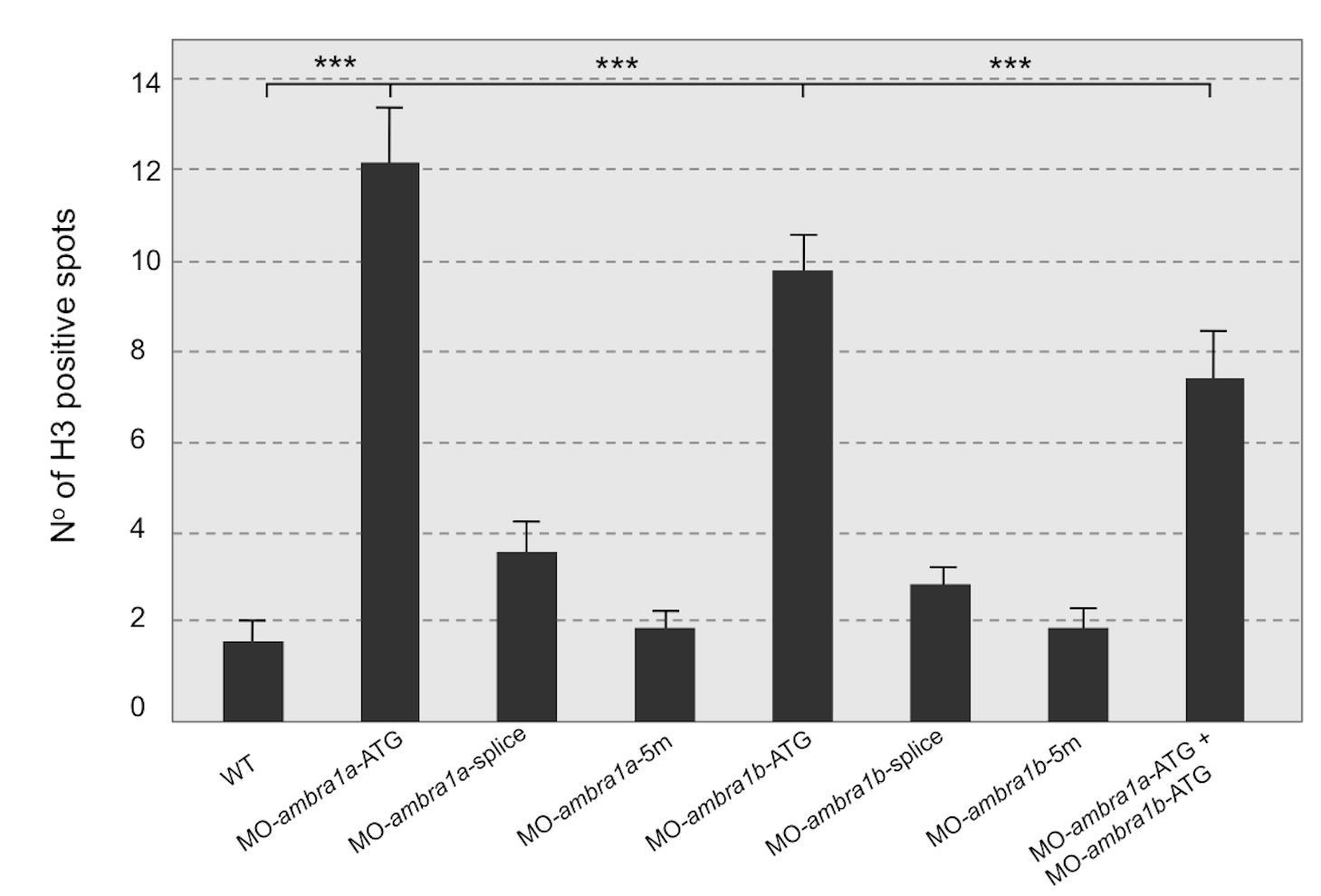

Supplement: Figure S5 — Analysis of the number of mitotic cells present in the same six somites region of 10 embryos of each category. Data are presented as the mean ±SEM. ***, P<0.001. (TIF) [file pone.0099210.s005.tif]

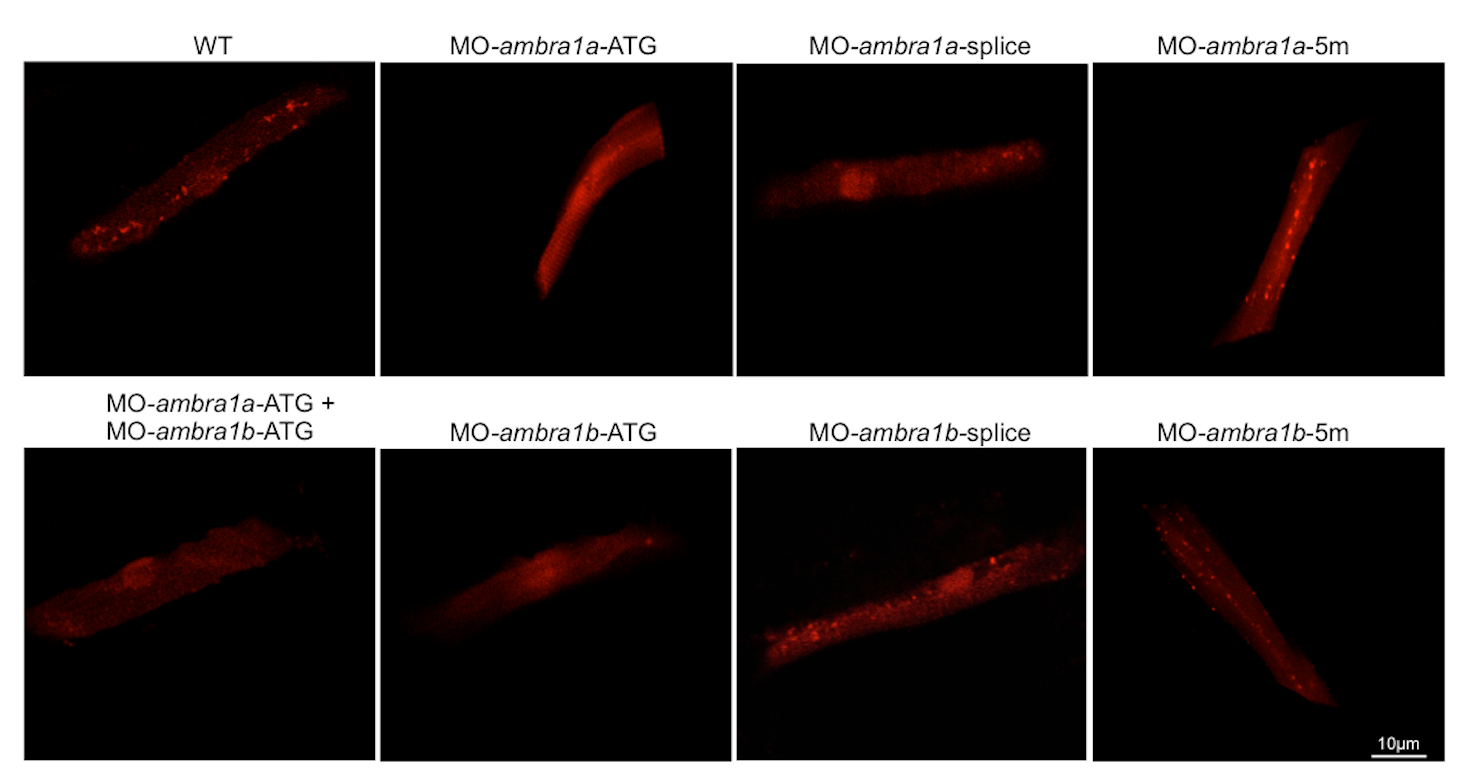

Supplement: Figure S6 — Fluorescence detection in muscle fibers of control and ambra1 morphant embryos following transfection with a Lc3-RFP reporter construct. Several fluorescent puncta have been detected in the transfected muscle fibers from WT and 5m-morphant embryos whereas only few puncta were visibile in ATG-morphant embryos. (TIF) [file pone.0099210.s006.tif]
